# Synthesis and Characterization of Nanoscale Tungsten Particles with Hollow Superstructure Using Spray Drying Combined with Calcination Process

**DOI:** 10.1186/s11671-019-2904-3

**Published:** 2019-02-26

**Authors:** Panchao Zhao, Wei Yi, Qigao Cao, Bosheng Zhang, Kunkun Chen, Rui Dang, Jialin Chen

**Affiliations:** 1 Northwest Institute for Nonferrous Metal Research, Xian, 710000 China; 2 State Key Laboratory of Advanced Technologies for Comprehensive Utilization of Platinum Metals, Kunming Institute of Precious Metals, Kunming, 650106 China

**Keywords:** Nanoscale tungsten powders, Hollow superstructure, High-pressure gas, Two-step calcination, Sinter

## Abstract

**Abstract:**

Nanoscale tungsten (W) powder is used in some special materials. In this study, a hollow superstructure W powder consisting of nanoparticles was synthesized by spray drying combined with two-step calcination from commercial (NH_4_)_6_W_7_O_24_·6H_2_O. The high-pressure gas (HPG) was the significant factor in spray drying process, which affect the BET surface area and average particles size of the spray-dried powders. The detailed influences of calcined steps and calcination temperature in the microstructure and average particles size of final W particles were investigated. The size distribution of as-synthesized nanoscale W particles with hollow superstructure was from 40 to 200 nm, and the average size was about 100 nm. The as-synthesized W powder shows good sintering properties. It should be noted that the powder technology in this study can be used to synthesize other powders with high-performance requirements.

**Graphical abstract:**

.
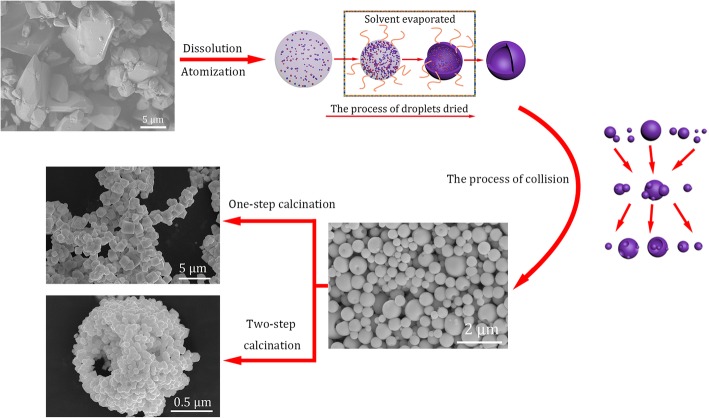

## Introduction

Tungsten (W) metal is widely used in daily life and modern industry because of its outstanding properties involving high melting point, high thermal conductivity, excellent mechanical properties at elevated temperatures, low sputtering yield, good mechanical strength, low tritium retention, and high erosion resistance [1–4]. The applications of W metal are also rapidly expanding in the account of increasing development of science and technology. Normally, W powders as raw material are selected to prepare products by powder metallurgical techniques, such as hot pressing (HP), hot isostatic pressing (HIP), and spark plasma sintering (SPS), so the performance of W particles is one of the critical factors to affect the properties of W products [5–7]. In general, there are many high requirements in W powders for preparing different products, such as the average particle size, distribution of particle size, surface area, and microstructure [8]. Many methods have been developed to synthesize W powders. Ren et al. [9] synthesized W particles with irregular morphology and very fine particle size of about 50 nm W by high-energy planetary milling. Tungsten compacts with relative density of 98.3% were achieved by the two-step sintered of W nanoparticles. Ryu et al. [10] prepared W powders with particle size less than 30 nm in a thermal plasma reactor. The relative density of W compact by sintered nanoscale W particles at 1400 °C was about 92%, while a lower relative density of about 72% was obtained by using micro-sized W particles. To develop high relative density tungsten compacts, suitable nanoscale W particles are desirable.

Compared with micro-sized W particles, nanoscale W particles have excellent superiorities due to their higher surface area and smaller grain, which can be sintered to W compacts with high relative density in relatively low temperature and low pressure [9–12]. Some methods of preparing nano-sized W particles were reported, including hydrogen reduction, sol-gel, and high-energy ball milling, and a plenty of useful data was obtained from previous study [9–15]. However, these techniques for synthesizing W nanoparticles still have some disadvantages, such as the multi-step high-temperature reduction process, burning of much energy, and using of expensive raw precursors. Hence, it is necessary to find an effective approach for preparing nano-sized W powder to achieve W sintered bodies with high relative density.

Spray drying technique is developed to be a clean, rapid, reproducible, low-cost, and easily scaled-up method to produce spherical nanoscale particles due to instantaneous transition between liquid (solvent) and solid (solute) phases [16–22]. Spray drying method compared with other drying techniques is widely used in industrial milling [16, 20]. It can synthesize sub-5 μm hollow and near-spherical superstructure particles consisted of nanoscale particles by self-assembly [16, 18]. During the process of spray drying, the solution (solute-solvent) is completely atomized and dispersed into chamber (hot gas) when interacted with the high-pressure gas at the nozzle. Afterward, fogdrops atomized are evaporated in the drying chamber and spherical or near-spherical solid particles are formed simultaneously [17, 22]. The particles prepared by spray drying have high dispersibility, good sphericity, and homogeneity. Moreover, different particle size distribution and microstructure of particles atomized can be obtained by controlling spray drying parameters [16, 18, 20, 22, 23]. In our previous study, high-performance ruthenium, micro-spherical tungsten-molybdenum alloy, and ruthenium compound particles were achieved by the spray drying process [21–23]. Therefore, the spray drying method can be chosen to synthesize nanoscale spherical W particles by optimizing spray drying parameters.

In this work, a systematic investigation was presented on the synthesis of nano-sized W particles with hollow superstructure by spray drying method combined with hydrogen reduction using ammonium paratungstate [(NH_4_)_6_W_7_O_24_·6H_2_O] as raw material. The influences of process parameters, including high-pressure gas of spray drying, heating rate of ignition, and reduction temperature, were discussed. The W compacts prepared by nano-sized W particles were also studied. To the best of our knowledge, there is no report on the synthesis of nano-sized W particles with hollow superstructure. The purpose of this research is to acquire the synthesis mechanism of nanoscale spherical particles with hollow superstructure and determine the best parameters by the investigation of parameters of spray drying and calcination process.

## Materials and Methods

Commercially available (NH_4_)_6_W_7_O_24_ ·6H_2_O powder was purchased from Xiamen Tungsten Co. Ltd. (Xiamen, Fujian, People’s Republic of China). The deionized water was used as the solvent, and all reagents and solvents were used as received without further purification. The (NH_4_)_6_W_7_O_24_·6H_2_O powder as a solute was dissolved in deionized water, and the solution was heated from room temperature to 80 °C for promoting dissolution. Then, the (NH_4_)_6_W_7_O_24_ solution with a certain concentration was obtained. The solution was atomized to micro-spherical particles by spray drying equipment with the optimizing parameters. Subsequently, the spray-dried (NH_4_)_6_W_7_O_24_ powder was ignited by two different calcined processes (the one-step direct calcination and the two-step calcination). Spray-dried (NH_4_)_6_W_7_O_24_ particles were heated to 800 °C for 60 min with the heating rate of 5 °C/min in N_2_/H_2_ (vol 5:5), which obtained cubic polyhedron W particles in the one-step calcination. The nanoscale near-spherical W particles with hollow superstructure were synthesized by the two-step calcination. The temperature went up to 650 °C for 20 min followed by cooling. Then, the temperature was reheated up to 700 °C for 120 min (heating rate of 5 °C/min in N_2_/H_2_ (vol 5:5)). As-synthesized W powder (two-step calcination) was uni-axially compacted to form pellets (10 mm diameter, about 4 mm height) under a pressure of 120 MPa without adding any external lubricant. The compact was sintered under H_2_ atmosphere for 2 h at 1400 °C (heating rate 20 °C/min).

A commercial spray drying equipment (B290, Buchi, Switzerland) was used to synthesize micro-spherical (NH_4_)_6_W_7_O_24_·6H_2_O particles. The solid phases in different stages were characterized by X-ray diffraction (XRD, Empyrean, PANalytical, the Netherlands, CuKα radiation at 40 kV). The microstructure of (NH_4_)_6_W_7_O_24_·6H_2_O particles and thermally decomposed products were observed by field emission scanning electron microcopy (FESEM, S-4800, Hitachi, Japan) and transmission electron microscopy (TEM, G2 20 200kV Tecnai). The average particle size, size distribution, and specific surface area of the as-prepared (NH_4_)_6_W_7_O_24_ powder were measured by the laser particle size analyzer (Mastersizer3000, Malvern, UK) and surface area instrument (Tristar II, Micromeritics, USA), respectively. The density of the sintered specimen was measured by Archimedes method. The hardness of as-sintered W specimen was measured by Vickers hardness tester (VMHT Auto, Leica, Germany).

## Results and Discussion

**Fig. 1:**
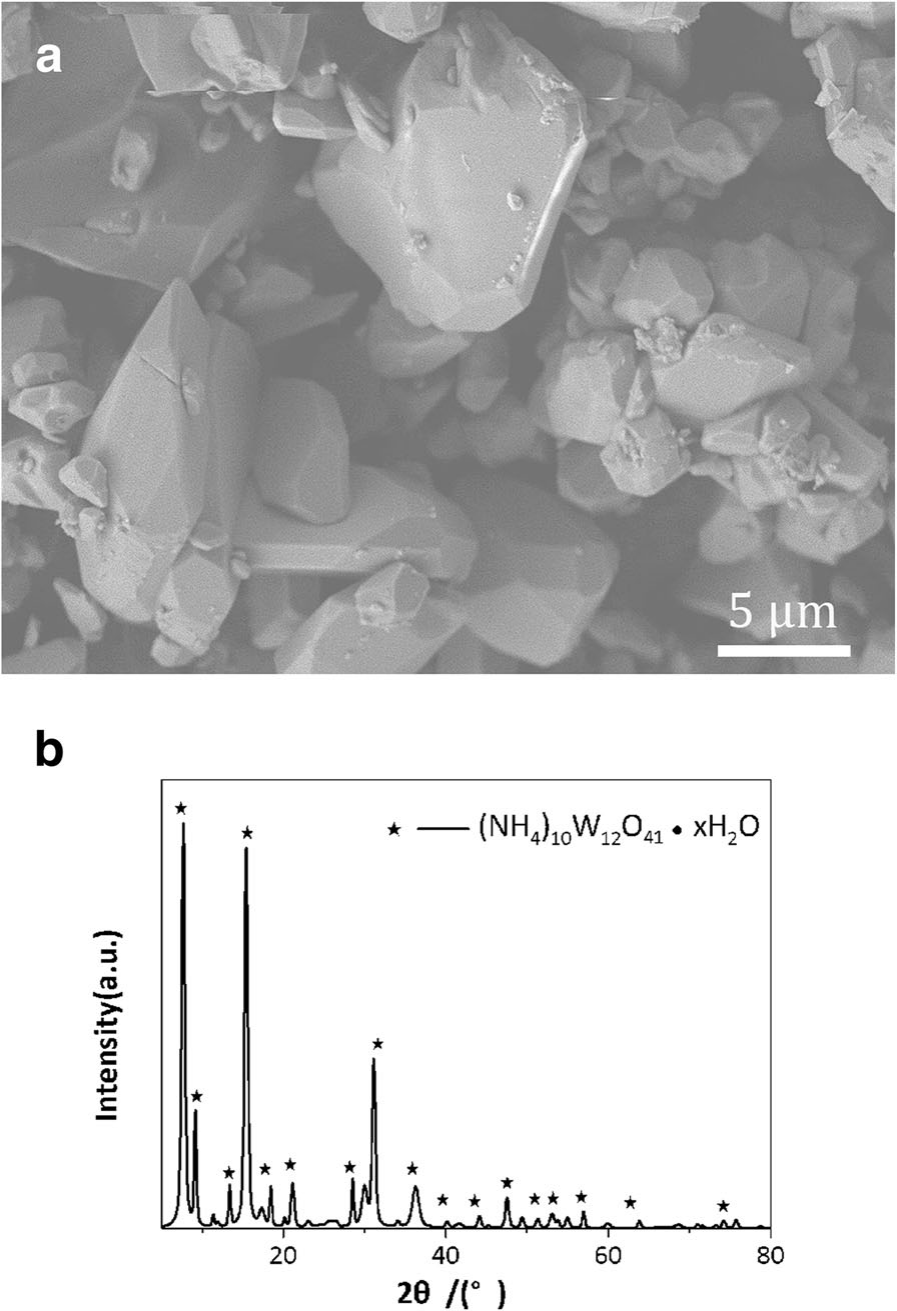
The microstructure (**a**) and XRD pattern (**b**) of commercially available (NH_4_)_6_W_7_O_24_·6H_2_O powders, or (**a**) the microstructure of commercially available (NH_4_)_6_W_7_O_24_·6H_2_O powders, and (**b**) the XRD pattern commercially available (NH_4_)_6_W_7_O_24_·6H_2_O powders

The microstructure of commercial (NH_4_)_6_W_7_O_24_·6H_2_O powder is presented in Fig. 1a. A lot of blocks composed of irregular micro-sized polyhedra can be observed. It is difficult to obtain near-spherical W powder with hollow superstructure consisted of nanoscale particles by direct ignition reduction with untreated (NH_4_)_6_W_7_O_24_·6H_2_O. So, the modification of microstructure of (NH_4_)_6_W_7_O_24_·6H_2_O is quite essential for the next step. The XRD pattern of untreated (NH_4_)_6_W_7_O_24_·6H_2_O is shown in Fig. 1b. All observed diffraction peaks observed can be matched in the International Centre for Diffraction Data (formerly Joint Committee on Powder Diffraction Standards JCPDS 7-240 database). So, the purchased powder is identified as (NH_4_)_6_W_7_O_24_·6H_2_O [24].

**Fig. 2:**
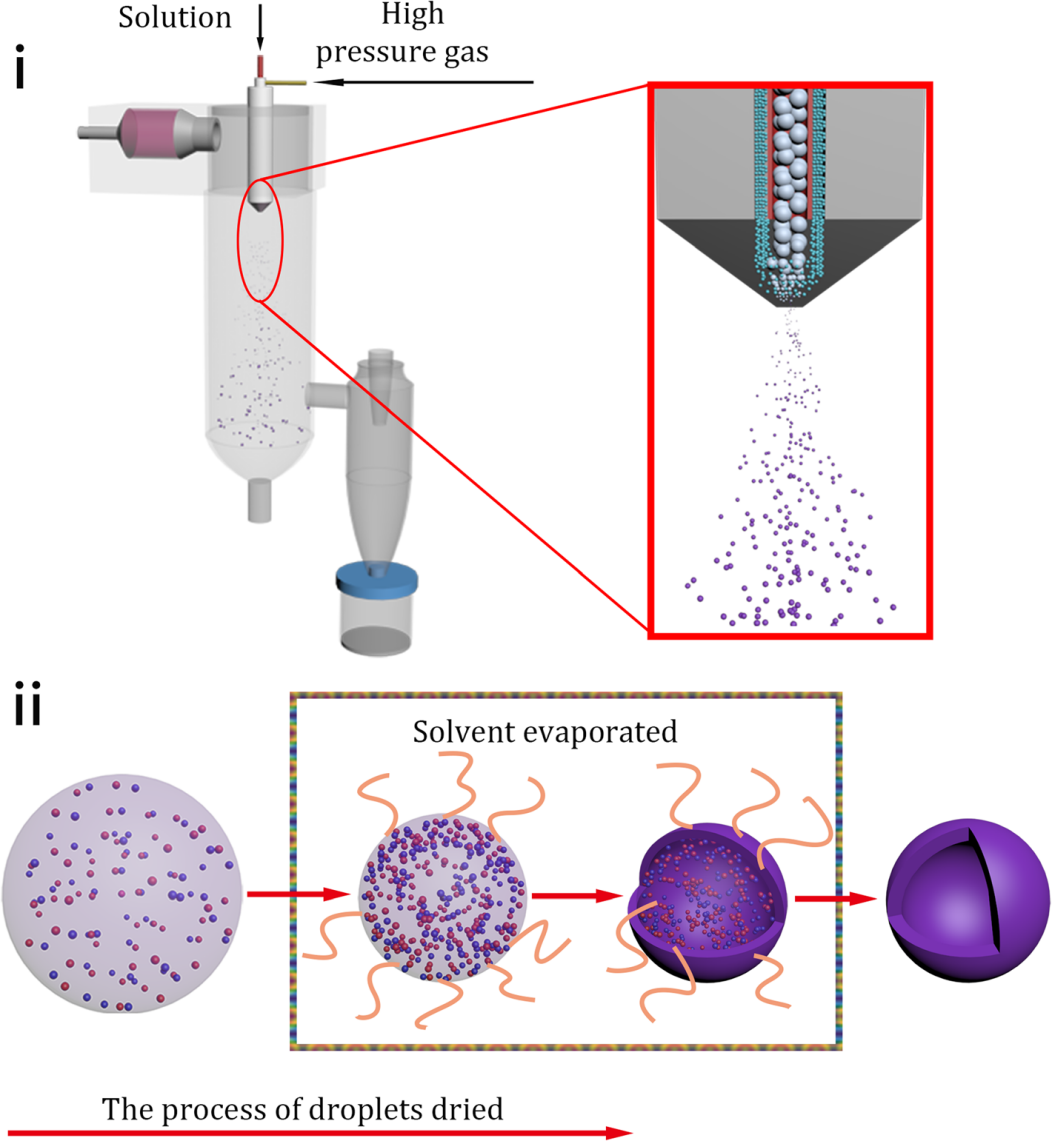
The schematic of spray drying process. **i** The detailed schematic of formed process of particles. **ii** The specific process of transformation of droplets

The detailed schematic of particles formed in spray drying is shown in Fig. 2i. The solution and high-pressure gas interact at the nozzle zone due to their high relative speed. Then, the solution is crashed into a lot of small fogdrops in the drying chamber. Subsequently, the atomized fogdrops are instantaneously dried to micro-sized spherical solid particles. The specific process of fogdrops transformation in the drying chamber is observed in Fig. 2ii. At the beginning, the volume of fogdrops became small because of evaporation of solvent (deionized water) from the surface of droplets, to increase the concentration of solute at fogdrops surface. Then, the solute starts to be separated out, forming solid (NH_4_)_6_W_7_O_24_ in the exterior of fogdrops. The volume of the fogdrops ceased to change when the concentration of solute at fogdrops surface reached saturation. After that, a shell formed on the surface of the fogdrops where the interior solute constantly formed into solid (NH_4_)_6_W_7_O_24_. The internal water vapor escaped from the shell to produce numerous nano-sized capillary pores which were hard to be observed. Finally, the micro-sized hollow spherical particles were obtained.

**Fig. 3:**
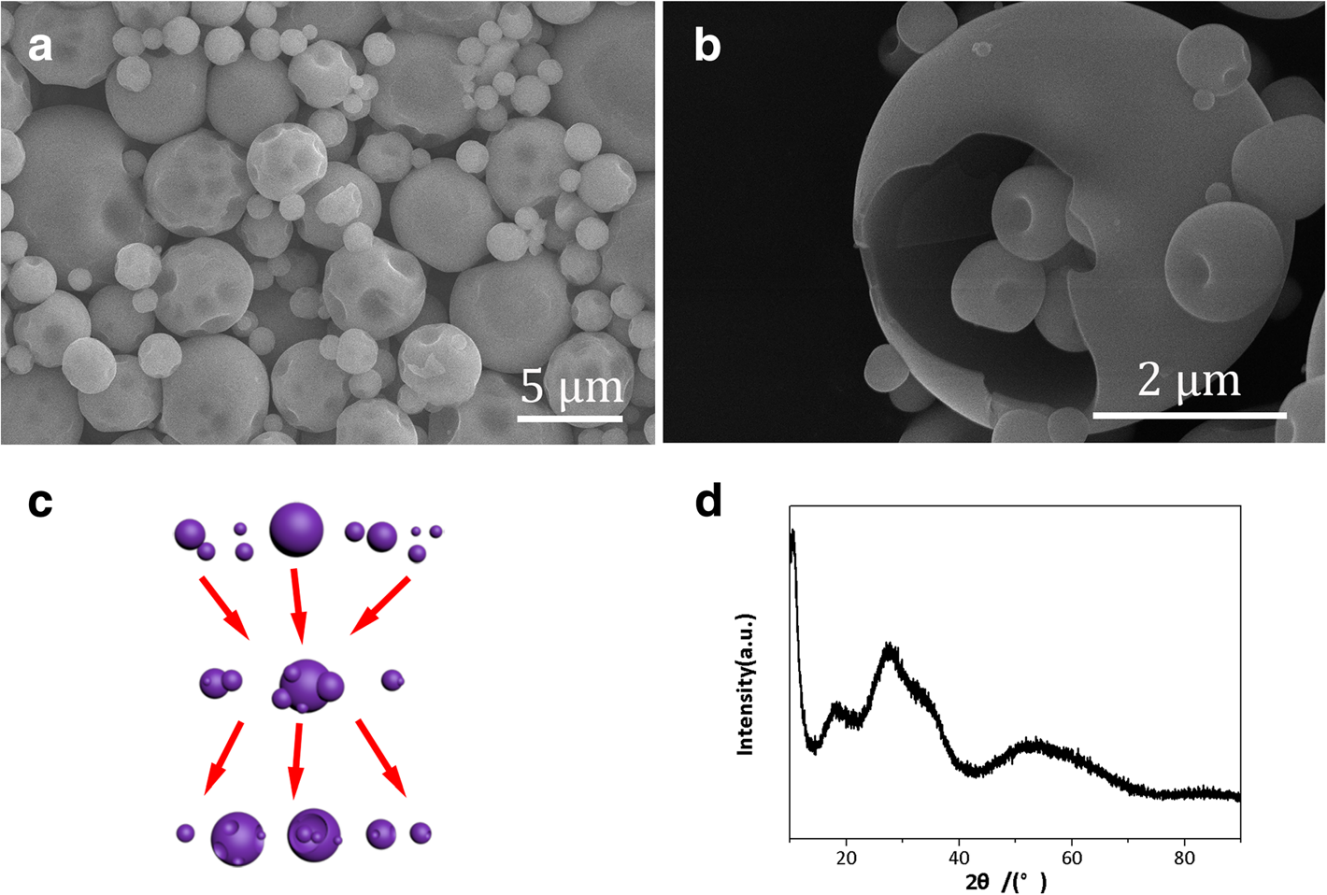
The microstructure and XRD pattern of spray-dried (NH_4_)_6_W_7_O_24_·6H_2_O particles. **a**, **b** The morphology of the (NH_4_)_6_W_7_O_24_·6H_2_O particles atomized by spray drying method. **c** The schematic of collision between particles. **d** The XRD pattern of spray-dried (NH_4_)_6_W_7_O_24_·6H_2_O powder

The microstructures of the (NH_4_)_6_W_7_O_24_ particles atomized by spray drying method are shown in Fig. 3a, in which nearly spherical and submicron particles are observed. There are numerous concaves on the surface of the sphere while the broken sphere is almost observed even in a miniscule amount in Fig. 3b. The detailed schematic of collisions between particles in Fig. 3c can provide a clue as to why the particles contain concaves and breaks. According to Fig. 3c, it is indicated that a large number of particles in the drying chamber can collide with each other due to the high-speed particles produced by high-pressure gas, and the main collision pattern is the bounced off each other resulted formation of concaves while the rare non-bounced collision causes the fracture of the sphere surface. The XRD pattern of spray-dried (NH_4_)_6_W_7_O_24_·6H_2_O is shown in Fig. 3d. Obviously, the amorphous peaks are observed, which indicates that the spray-dried particles are completely noncrystalline substance. It is quite likely because a very short drying time (approximately 0.2 s) of each fogdrops cannot provide enough time for crystallization.

**Fig. 4:**
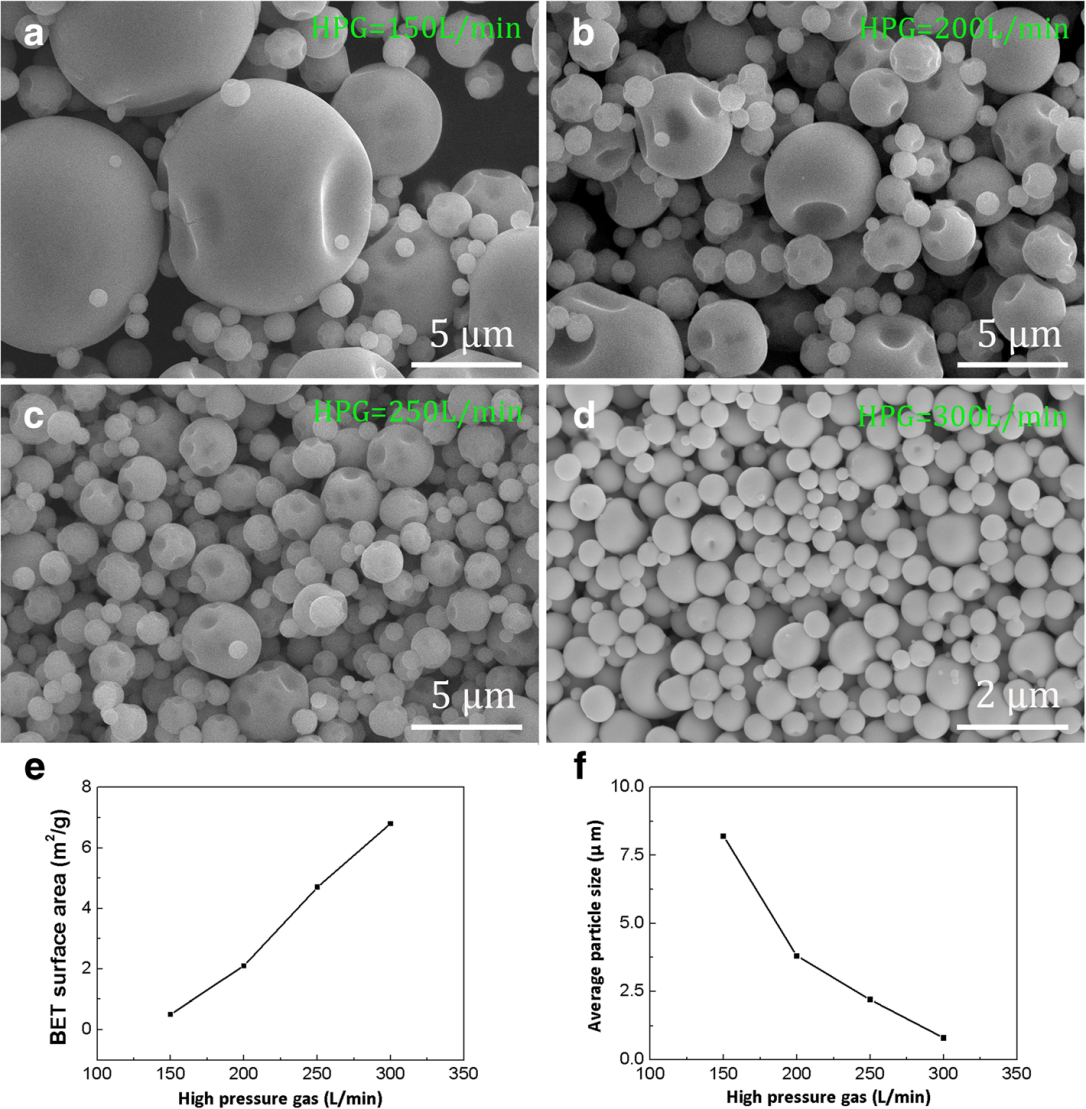
The microstructure of spray-dried (NH_4_)_6_W_7_O_24_·6H_2_O particles in different high-pressure gas. **a** 150 L/min. **b** 200 L/min. **c** 250 L/min. **d** 300 L/min. **e** BET surface area. **f** Average particle size

The microstructures of spray-dried (NH_4_)_6_W_7_O_24_ particles in different high-pressure gas (HPG) are shown in Fig. 4, where significantly different microstructures of (NH_4_)_6_W_7_O_24_ particles are observed. The BET surface area and average particle size of as-prepared (NH_4_)_6_W_7_O_24_ powders are also presented in Fig. 4. During spray drying, the HPG as another critical parameter which can influence the microstructure and particle size distribution was investigated, and the drying temperature, solution concentration, and gas/liquid rates were studied in our previous work [21, 22]. In this part of the experiment, the drying temperature, solution concentration, and gas/liquid rates were controlled at 210 °C, 0.01 mol/L and 5 nL/5 mL, respectively. When the HPG was 150 L/min, submicron nearly spherical (NH_4_)_6_W_7_O_24_ particles without any agglomeration were obtained (Fig. 4a). The BET surface area and average particle size (in Fig. 4e, f) were circa 0.5 m^2^/g and 8.3 μm, respectively. The distribution range of particle size distribution was quite large (Fig. 4a). When the HPG got to 200 L/min, the surface features were almost the same as that at 150 L/min. However, the BET surface area and average particle size were about 2.1 m^2^/g and 3.8 μm, respectively, which were obviously different from those at 150 L/min. The distribution range of particles size distribution was declined slightly, as shown in Fig. 4b, e, and f. In Fig. 4c, e, and f, when the HPG went up to 250 L/min, the characteristics of particles were changed except the surface features. And the BET surface area increased to about 5.1 m^2^/g, the average particle size reduced to approximately 2.1 μm, and the range of particles size distribution became very narrow. When the HPG reached the maximum value of the spray drying device (300 L/min), the concaves on the exterior of particles were noticeably reduced (Fig. 4d). The BET surface area and average particle size were about 6.8 m^2^/g and 0.9 μm, respectively. The range of particles size distribution range was quite small as shown in Fig. 4 e, f.

The HPG is one of the most important factors in atomization process, which can influence the BET surface area and average particle size of spray-dried particles. When the HPG was 150 L/min, the (NH_4_)_6_W_7_O_24_ solution at the nozzle was not sufficiently crashed into micro-sized fogdrops by HPS owing to the low relative speed between solution and HPG. As a result, the dried powders had larger average particles size and lower BET surface area (Fig. 4a, e, f). On the other hand, when the HPG was adjusted to the largest (300 L/min), the solution of (NH_4_)_6_W_7_O_24_ was completely transformed into ultrafine fogdrops because of a high relative speed between them, so the spray-dried particles had a small average size (0.9 μm) and a large BET surface area (6.8 m^2^/g), as shown in Fig. 4d, e, f. Consequently, the larger average (NH_4_)_6_W_7_O_24_ particles with lower BET surface area could be acquired by using a small HPG. The result could be the opposite when the HPG were larger.

**Fig. 5:**
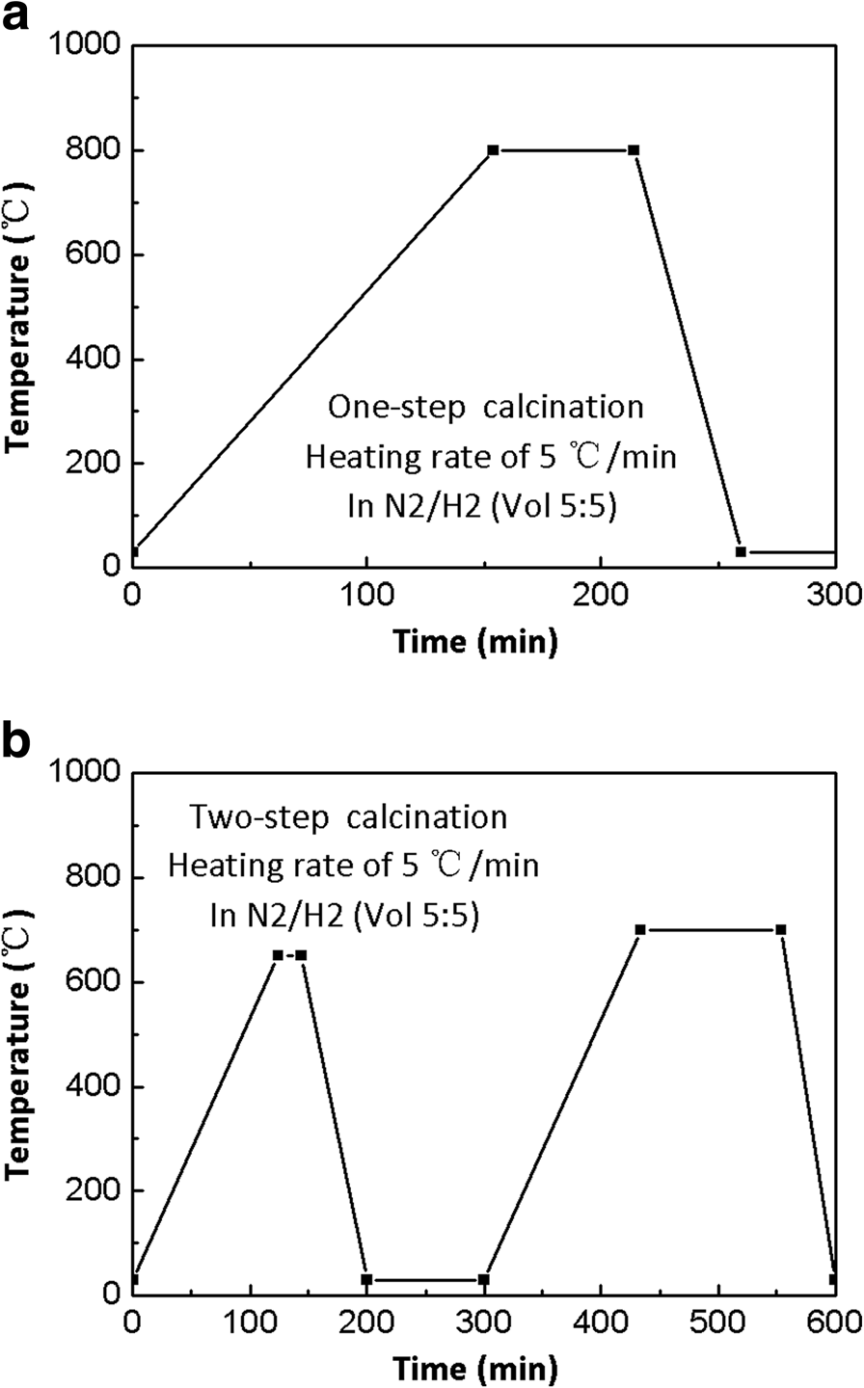
The two different reduction calcination processes of spray-dried (NH_4_)_6_W_7_O_24_·6H_2_O particles. **a** One-step direct calcination. **b** Two-step calcination

**Fig. 6:**
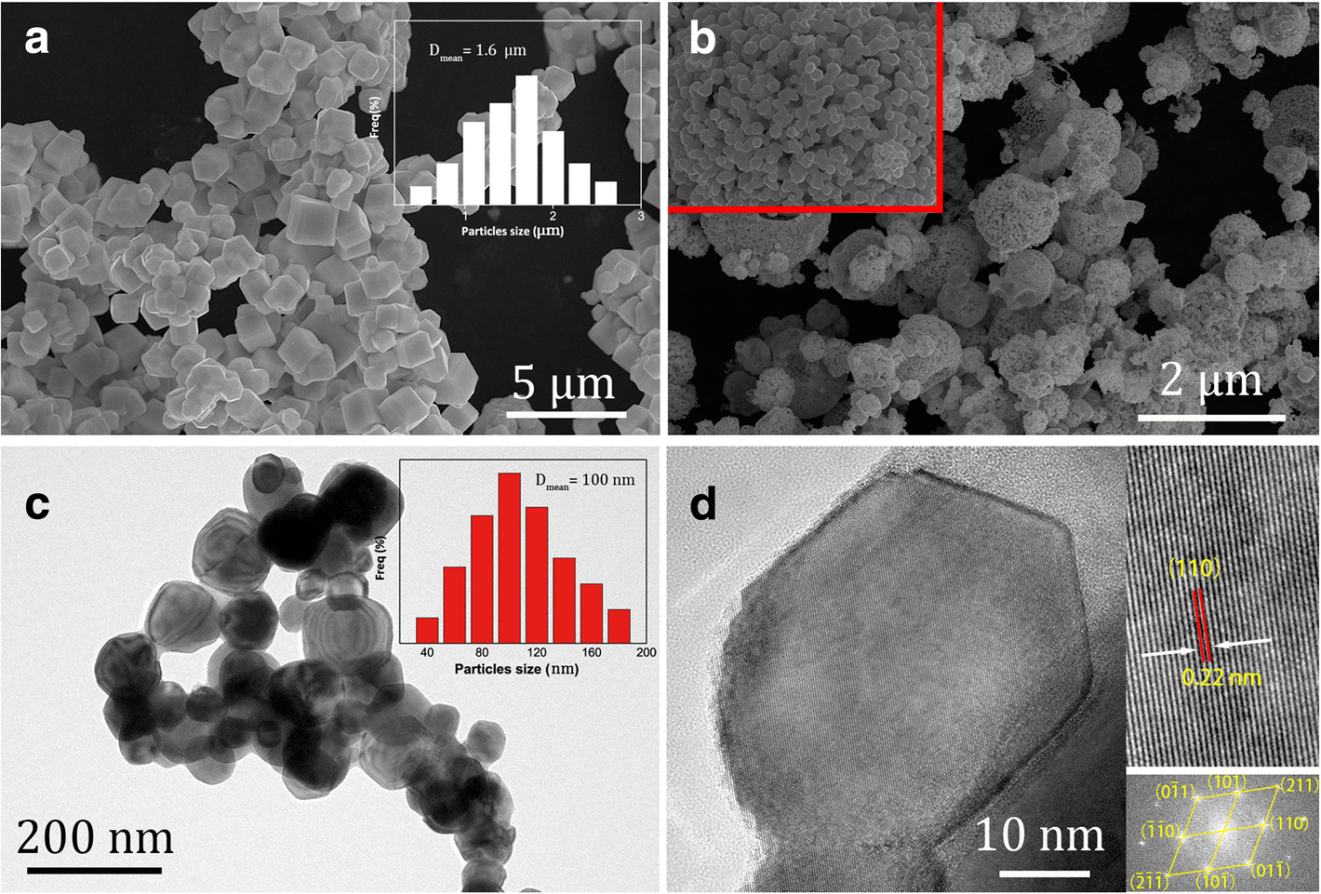
Characterization of W powders. **a** Microstructure of W particles prepared by one-step calcination. **b** Microstructure of W particles prepared by two-step calcination. **c** TEM image of W particles with particle size distribution by two-step calcination and **d** HRTEM image of W particles with SAED pattern inset by two-step calcination

In this work, spray-dried (NH_4_)_6_W_7_O_24_ particles (HPG was 300 L/min) were calcined by two different reduction calcination processes. The one-step direct calcination was carried out, as shown in Fig. 5a. The microstructures of W particles are presented in Fig. 6a, in which cubic polyhedron W particles with average size of 1.6 μm are observed. On the one hand, the nanoscale near-spherical W particles with hollow superstructure cannot be obtained by one-step direct calcination. On the other hand, the two-step calcination was illustrated in detail in Fig. 5b. The microstructures of W powders are shown in Fig. 6b, and the hollow superstructure W consisting of nanoparticles are observed in the insert of Fig. 6b. The TEM photographs of W nanoparticles are shown in Fig. 6c. The near-spherical particles are observed, and the average particle size is about 100 nm. The lattice structure and the diffraction pattern of nano-sized W particles are observed in Fig. 6d, and the interplanar spacing of nano-sized W was determined to be about 0.22 nm. According to the interplanar spacing and the diffraction pattern in the illustration of Fig. 6d, it is confirmed that the nanosized particles are single-crystal W [25].

**Fig. 7:**
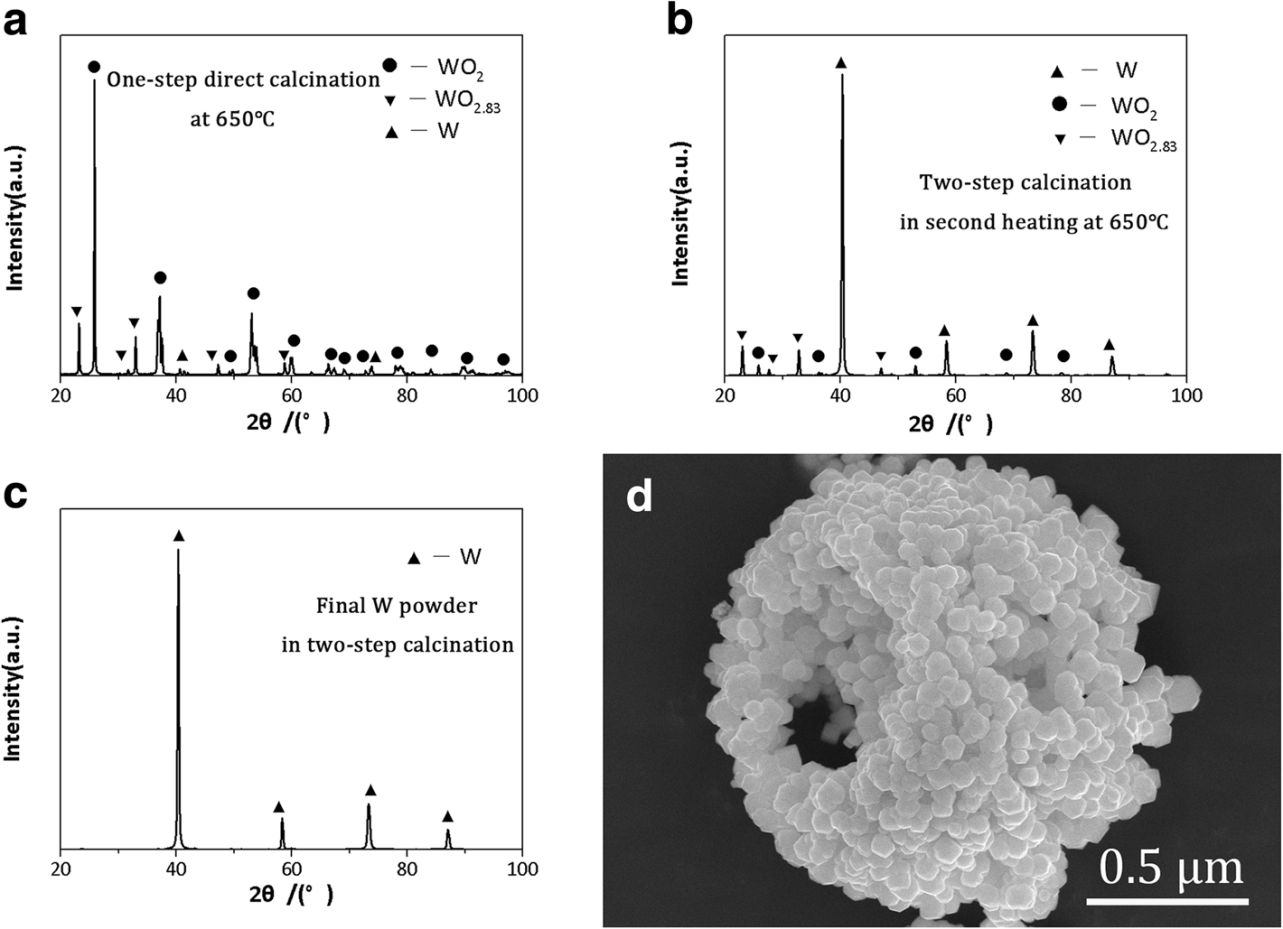
The XRD pattern and microstructure of products. **a** The XRD pattern of intermediates in one-step direct calcination at 650 °C. **b** The XRD pattern of intermediates in two-step calcination in second heating at 650 °C for 20 min. **c** The XRD pattern of final W powder in two-step calcination. **d** The microstructure of final W powder in two-step calcination

The different results of W particles were caused by two different calcined processes. In the one-step direct calcination process, when the temperature was heated to 650 °C, the new W-phase was generated and weak, as shown in Fig. 7a. The newborn W particles were easily agglomerated due to their high activity and energy. When the temperature continued to rose up to 800 °C, the W particles grew up rapidly. It should be noted that WO_*x*_ is easy to sublimate at 720 °C or higher temperature [26], and WO_*x*_ sublimation can promote W shift to fall on the newly formed W particles, which lead to the growth of W particles. As a result, the lager W particles with an average size of 1.6 μm were obtained (in Fig. 6a).

**Fig. 8:**
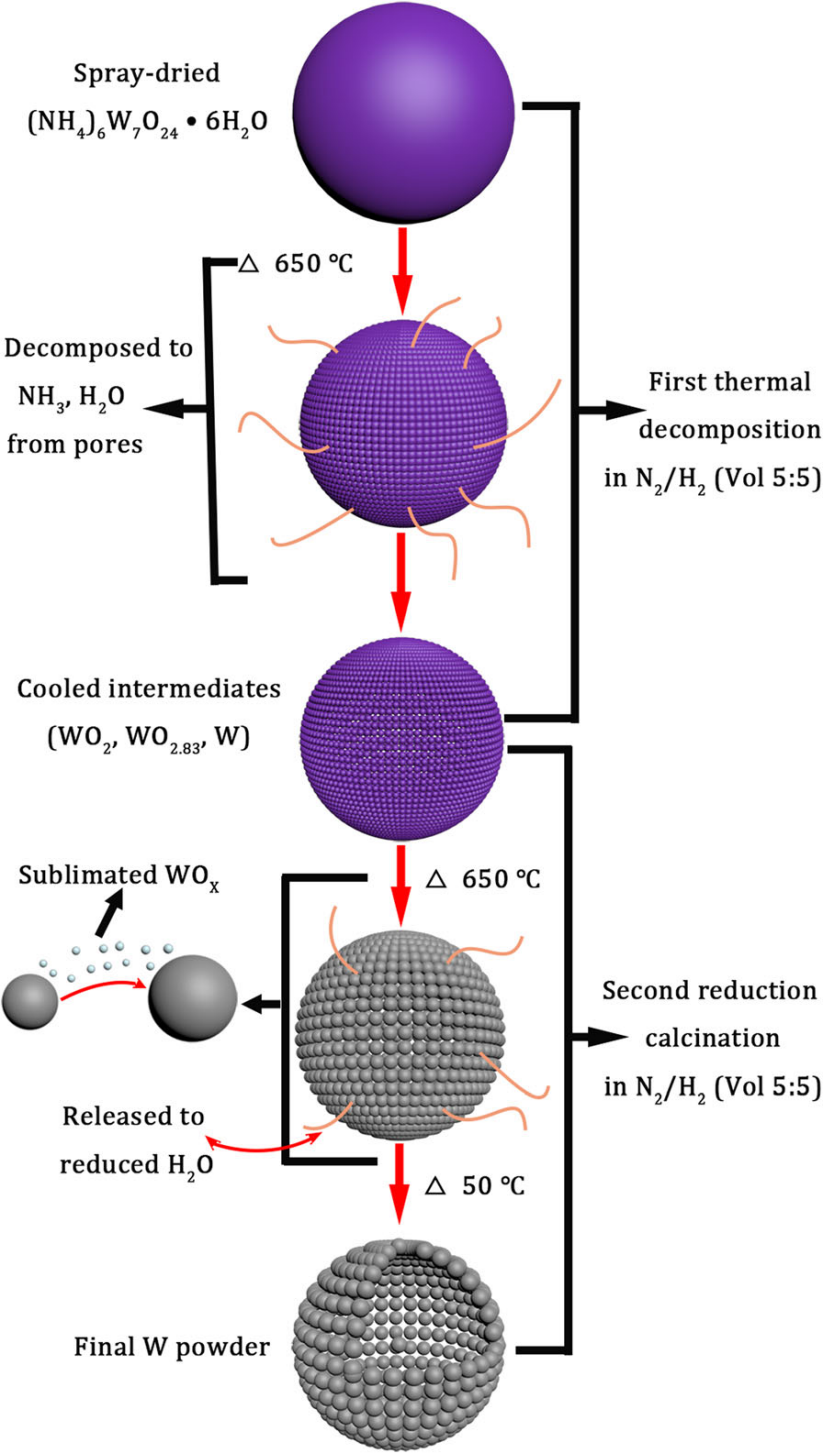
The microstructural transformation mechanisms of spray dried (NH_4_)_6_W_7_O_24_·6H_2_O particles reduction calcination

In the two-step calcination process, the detailed formation mechanism of hollow W nanoparticles is shown in Fig. 8. Firstly, when the temperature was heated to 650 °C for 20 min followed by cooling, spray-dried (NH_4_)_6_W_7_O_24_ decomposed to NH_3_, H_2_O, and intermediates (WO_2_, WO_2.83_, W). The newborn W had low activity and energy because of cooling, which had a restriction on the growth of W particles. When the intermediates were reheated to 650 °C, the W particles started to increase slightly because of a few WO_*x*_ sublimated, and the XRD patterns of intermediate products are shown in Fig. 7b. When the temperature of reduction calcination was increased to 700 °C, the gases (N_2_/H_2_) were easy to enter into the pores between W nanoparticles, which could increase the reaction rate and contaction between gases and intermediates. The sublimated WO_*x*_ decreased obviously at 700 °C due to the sublimation of WO_*x*_ at 720 °C or higher temperature [26]; thus, the hollow superstructure W composing of W nanoparticles could be obtained. The XRD patterns and microstructure of the final W powders are presented in Fig. 7c and Fig. 7d, respectively.

**Fig. 9:**
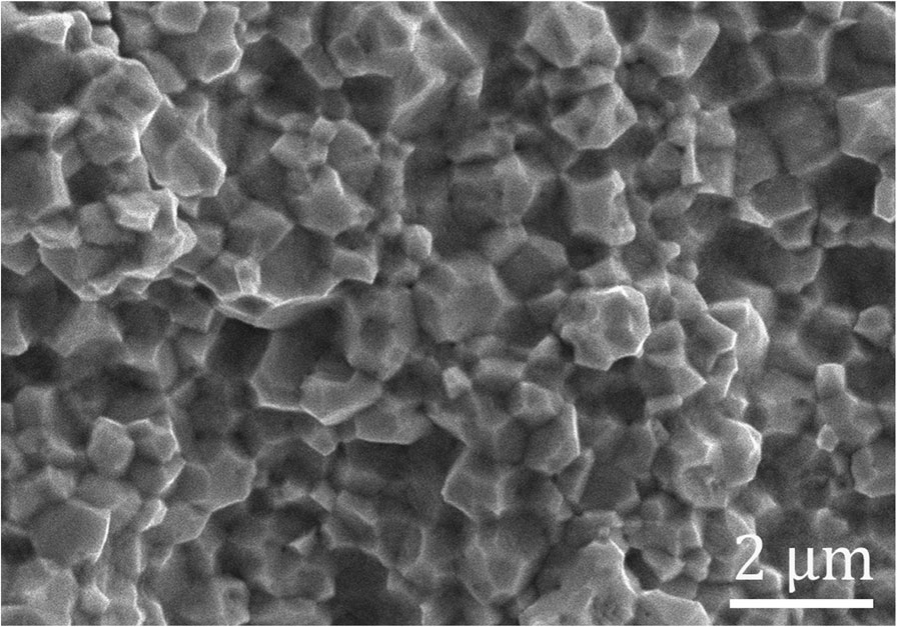
The microstructure of the W compact

As-synthesized W powder (two-step calcination) was sintered at 1400 °C. The microstructure of W compact is shown in Fig. 9, where uniform granular structure with polyhedral shapes and defined grain boundaries is observed. The density of as-sintered W specimen, 18.97 g/cm^3^, was obtained, and this corresponds to a relative density of 98.55% based on the tungsten theoretical density. Moreover, Vickers hardness of as-fabricated W compact was 384 ± 10 Hv. It is indicated that the W powder prepared by spray drying and two-step reaction has good sintering property.

## Conclusions

The hollow superstructure W powders composed of nanoparticles were synthesized by spray drying combined with two-step calcination from commercial (NH_4_)_6_W_7_O_24_·6H_2_O. The three-dimensional irregular polyhedral (NH_4_)_6_W_7_O_24_·6H_2_O was successfully turned into the micro-sized near-spherical particles by spray drying method, from which the high-pressure gas (HPG) was one significant factor to synthesized near-spherical (NH_4_)_6_W_7_O_24_ powders. When the HPG was 300 L/min, well near-spherical particles with a small average size (0.9 μm) and large BET surface area (6.8 m^2^/g) could be obtained. Different reduction calcination process had great influence on the microstructures of W particles. The cubic polyhedron W particles with average size of 1.6 μm were synthesized by one-step reduction calcination. In the two-step reduction calcination process, the hollow superstructure W composed of nanoparticles were obtained. The particle size distribution of as-synthesized W powders by two-step reduction calcination ranged from 40 to 200 nm, and the average particle size was about 100 nm. W target was acquired by sintering hollow superstructure W powders. The relative density and Vickers hardness were 98.55% and 384 ± 10 Hv, respectively. The as-synthesized W powder showed good sintering properties. The powder technology in this study can be applied to synthesize other powders with high-performance requirements.

## Abbreviations

BET: Brunauer, Emmett and Teller
FESEM: Field emission scanning electron microcopy
HIP: Hot isostatic pressing
HP: Hot pressing
HPG: High-pressure gas
SAED: Selected area electron diffraction
SPS: Spark plasma sintering
TEM: Transmission electron microscopy
W: Tungsten
XRD: X-ray diffraction
