# Correction to: Synthesis and Characterization of Nanoscale Tungsten Particles with Hollow Superstructure Using Spray Drying Combined with Calcination Process

**DOI:** 10.1186/s11671-019-3173-x

**Published:** 2019-10-18

**Authors:** Panchao Zhao, Wei Yi, Qigao Cao, Bosheng Zhang, Kunkun Chen, Rui Dang, Jialin Chen

**Affiliations:** 1 Northwest Institute for Nonferrous Metal Research, Xian, 710000 China; 2 State Key Laboratory of Advanced Technologies for Comprehensive Utilization of Platinum Metals, Kunming Institute of Precious Metals, Kunming, 650106 China

**Correction to: Nanoscale Res Lett**

**https://doi.org/10.1186/s11671-019-2904-3**

In the article [1], the use of the formula (NH_4_)_6_W_7_O_24_·6H_2_O to represent the starting material ammonium paratungstate (APT) is outdated and incorrect.

The correct version of the formula for APT is (NH_4_)_10_[H_2_W_12_O_42_]·*n*H_2_O (*n* = 4, 10, 6).

The authors thank the reader who brought this to their attention after their article was published.
